# The childhood arthritis & rheumatology research alliance network registry: demographics and characteristics of the initial 6-month cohort

**DOI:** 10.1186/1546-0096-10-S1-A57

**Published:** 2012-07-13

**Authors:** Marc D Natter, Jane R Winsor, Kathleen A Fox, Norman T Ilowite, Kenneth D Mandl, Kelly L Mieszkalski, Christy I Sandborg, John S Sundy, Carol A Wallace, Laura E Schanberg

**Affiliations:** 1 Children's Hospital Boston, Boston, MA, USA; 2 Children's Hospital Montefiore, Bronx, NY, USA; 3 Duke Clinical Research Institute, Durham, NC, USA; 4 Duke University Medical Center, Durham, NC, USA; 5 Seattle Children's Hospital & Regional Medicine, Seattle, WA, USA; 6 Stanford Medical Center, Stanford, CA, USA; 7 Stanford, CA, USA

## Purpose

In 2009, the Childhood Arthritis and Rheumatology Research Alliance (CARRA) established a longitudinal multi-center, multiple disease U.S. national registry (CARRAnet) for pediatric rheumatology with the intent of providing 60 participating clinical sites a new framework to drive observational clinical research and evidence-based care. CARRAnet seeks to enroll up to 20,000 subjects with childhood-onset rheumatic disease and twice yearly follow-up. We report baseline characteristics of the initial 6-month enrollment cohort; disease-specific results are reported separately.

## Methods

Enrollment commenced 5/29/2010 with data available through 12/28/10. Inclusion criteria comprised 1 of 8 categories of defined rheumatic disease with onset before the 16^th^ birthday in subjects <=21 years (localized scleroderma, juvenile dermatomyositis, juvenile idiopathic arthritis, juvenile primary fibromyalgia syndrome, SLE or mixed connective tissue disease, sarcoidosis, systemic sclerosis, and vasculitis). A common baseline data set and 1 disease-specific data set was completed on each participant by interview and chart review. Data cleaning and analysis employed Microsoft Excel and Access (Microsoft Corp), SAS (SAS Institute), and R (R Foundation for Statistical Computing).

## Results

1638 subjects were enrolled from 27 centers throughout the US. The analysis cohort reflected 1371 subjects, predominantly JIA; 63 variables were collected for the shared baseline form with summary statistics presented in the figures. The population reported overall good to excellent health by patient and physician report: 96% with mean HRQOL good to excellent; physician mean global assessment of disease activity (PGAS) 1.6 (0-10 scale). PGAS correlated with subject reports (CHAQ, subject global, subject pain scores – Pearson corr 0.33, 0.39, 0.42 respectively). Medication use was prevalent, including 74% ever on steroids, 41% ever on biologics, and 31% currently on biologics. Growth was within normal on average, but exhibited wide deviation (-5.3>weight Z > 4.7, -19.7>height Z>9.4). A similarly wide range was seen on both objective and subjective measures, identifying probable subpopulations with high disease activities.

**Table 1:** Summary characteristics of CARRAnet initial cohort

Demographic measures	N (%)
Total participants studied	1371
Female gender	1024 (75%)
White/caucasian	1187 (87%)
Age at onset of symptoms, mean in years	7.2
Age at baseline visit, mean in years	11.8
Black or African American	127 (9%)
Asian	45 (3%)
American Indian or Alaska Native	30 (2%)
Native Hawaiian or Pacific Islander	9 (<1%)
Hispanic ethnicity	150 (11%)

**Primary rheuamtic diagnosis**	
Juveline idiopathic arthritis	1051 (77%)
Juveline dematophyositis	109 (8%)
Systemic lupus erythematosus	100 (7%)
Localized scleroderma	43 (3%)
Mixed connective tissue disease	23 (2%)
Vasculitis	23 (2%)
Systemic sclerosis	9 (<1%)
Sarcoidosis	8 (<1%)
Juveline primary fibromyalgia syndrome	6 (<1%)

**Growth parameters**	**Mean & (Range)**
Weight Z-score	0.28 (-5.3 – 4.7)
Height Z-score	-0.14 (-19.7 – 9.4)
BMI Z-score	0.38 (-5.0 – 4.1)

**Assessments (scale: best -> worst)**	**Mean & (Range)**
Physician global assess disease activity (0 – 10)	1.6 (0 – 10)
Subject global assess disease activity (0 – 10)	2.3 (0 – 10)
Subject pain score (0 – 10)	2.4 (0 – 10)
CHAQ (0 – 3)	0.33 (0 – 3)
HRQOL – Good, very good, excellent (%)	96%
HRQOL – Very poor, poor (%)	3%

**Family history (first degree relative) of**	**N (%)**
Psoriasis	82 (6%)
Rheumatoid arthritis	71 (5%)
Autoimmune throiditis	62 (5%)
Fibromyalgia	61 (4%)
Juvenile idiopathic arthritis	55 (4%)
Diabetes type I	35 (3%)
Inflammatory bowel disease	31 (2%)
Systemic lupus erthematosus	28 (2%)
Spondyloarthritis or ankyl. spondylitis	16 (1%)
Multiple sclerosis	13 (1%)
Celiac disease	10 (1%)
Uveitis	5 (<1%)

**Medication use**	**N (%)**
Steroids (ever)	1010 (74%)
Steroids (longterm daily, ever)	644 (47%)
Steroids (IV pulse, ever)	212 (15%)
Steroids (IA, ever)	529 (39%)
Biologics (ever)	560 (41%)
Biologics (current)	429 (31%)
TNF-α Blockers (current)	338 (25%)
DMARDs (ever)	1107 (81%)
DMARDs (current)	1021 (75%)
Methotrexate (current)	620 (45%)
NSAIDs (current)	625 (46%)
Opioids (current)	17 (1%)

**Figure 1:**
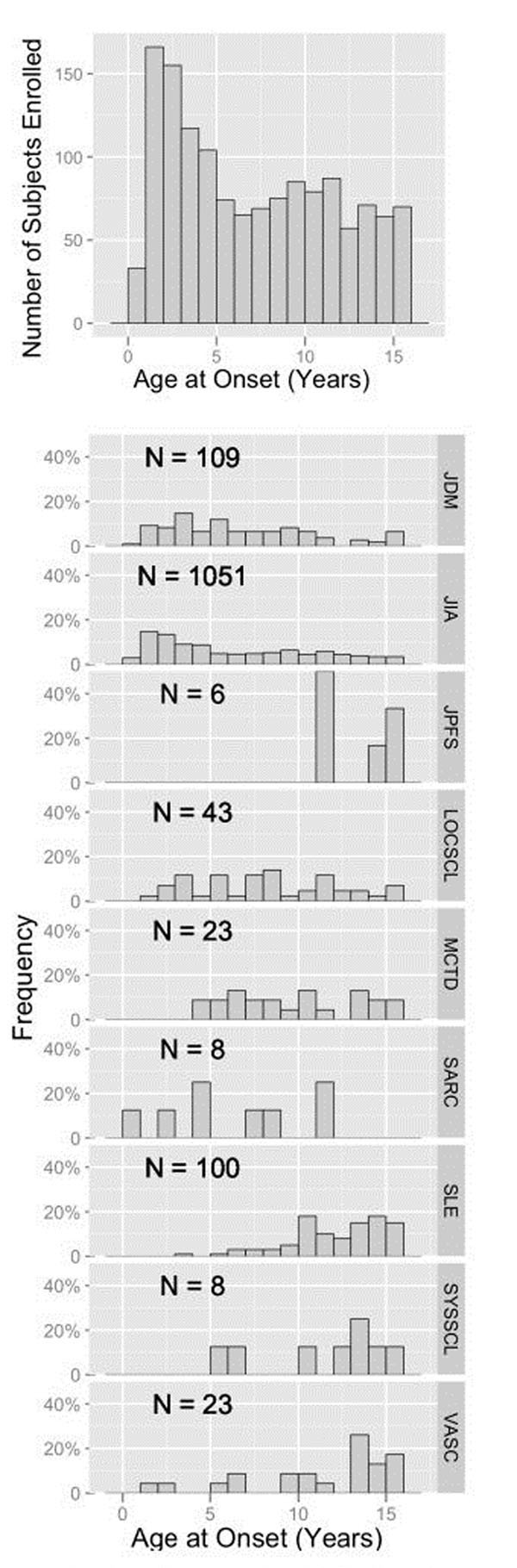
Disease onset distributions

## Conclusion

The initial CARRAnet cohort reflects predominantly low disease activity with favorable self-reports. This is not a population study and issues of enrollment bias require further investigation. Despite the overall well-being of the population, the high use of steroids, biologics, and DMARDs, along with significant subpopulations concerning for high disease activity, are important areas of future focus.

## Disclosure

Marc D. Natter: None; Jane R. Winsor: None; Kathleen A. Fox: None; Norman T. Ilowite: None; Kenneth D. Mandl: None; Kelly L. Mieszkalski: None; Christy I. Sandborg: None; John S. Sundy: None; Carol A. Wallace: None; Laura E. Schanberg: None; CARRAnet Investigators Group: None.

